# Role of microbial communities and nitrogen sources in suppressing root rot disease during ginseng cultivation

**DOI:** 10.3389/fmicb.2024.1396686

**Published:** 2024-07-04

**Authors:** Gyeongjun Cho, Da-Ran Kim, Youn-Sig Kwak

**Affiliations:** ^1^Division of Agricultural Microbiology, National Institute of Agricultural Sciences, Rural Development Administration, Wanju, Republic of Korea; ^2^Division of Applied Life Science and RILS, Gyeongsang National University, Jinju, Republic of Korea

**Keywords:** *Fusarium*, microbiota, monoculture, Pseudomonadaceae, suppressive soil

## Abstract

Ginsengs, widely acknowledged for their health-promoting properties, are predominantly grown for their roots, necessitating an extended cultivation period of a minimum of 4 to 6 years for maturation. The prolonged growth duration in a specific location makes ginseng plants susceptible to soil-borne ailments, such as root rot, leading to significant detrimental effects. Focusing on the crucial role of the plant microbial community in maintaining ginseng health, the study reveals that repeated and continuous cultivation leads to the collapse of the initial disease-suppressive rhizosphere community, resulting in severe root rot. The dominance of Pseudomonadaceae in the rhizosphere subsequently reinstates disease suppression, aligning with suppressive soil generation phenomena. The research investigates the applicability of identified patterns to field conditions and demonstrates that rhizosphere samples from the field closely resemble conditions observed in pot-based NH_4_Cl treatment experiments. These findings emphasize the critical role of the rhizosphere microbial community in ginseng health maintenance during extended cultivation, offering insights into disease prevention strategies. The study also suggests the potential of pot-based experiments in simulating field conditions and informs future approaches for sustainable ginseng cultivation.

## Introduction

The concept of the “rhizosphere,” originally introduced by Hiltner (1904), designates the region surrounding plant roots, where a dynamic interplay unfolds among microorganisms activated by the presence of these roots. Pinton et al. (2007) further delineated this as the habitat where rhizobacteria undergo colonization by root exudates. Within this intricate microbial tapestry of the rhizosphere, bacteria orchestrate several interactions that significantly impact various facets of plant biology, including growth, nutrition, and overall quality (Berg and Smalla, 2009). Of particular interest among these interactions are those that confer benefits to plants, leading to the emergence of plant growth-promoting rhizobacteria (PGPR). The well-documented *Bacillus* spp. and *Pseudomonas* spp. are prominent among the PGPR community and are recognized for their capacity to induce systemic resistance in plants (Guo et al., 2019). Consequently, they have garnered substantial attention as potential candidates for application as biofertilizers and biocontrol agents.

In addition to the rhizosphere, another crucial phenomenon that warrants exploration is the concept of “disease-suppressive soil” (Weller et al., 2002). This unique type of soil provides a natural shield, where the microbial community actively safeguards plants against common pathogens. Disease-suppressive soil can be classified into two distinct categories: general suppression and specific suppression (Schlatter et al., 2017). General suppression is mediated by non-specific mechanisms, such as trophic competition and interactions within the entire microbial community. Conversely, specific suppression is driven by individual microorganisms or select groups of microorganisms (Cook, 2014). Remarkably, specific suppressive soil can retain its protective properties even when diluted to as low as 1–10% of the total soil volume, highlighting its potential for transplantation and sustainable disease management (Weller et al., 2002).

The historical significance of ginseng (*Panax ginseng*), a medicinal herb native to Northeast Asia (Baeg and So, 2013), in oriental medicine dates back to ancient times, with its initial cultivation practices dating to approximately the 13th century around the region of Mt. Mohusan (35°02′02.9 “N 127°11′01.3”E) in Korea (Korean Culture and Information Service, 2014). Ginseng is renowned for its rich composition of active ingredients, including steroidal saponins, protopanaxadiols, protopanaxatriols, and other bioactive compounds, all of which have been documented to confer various health benefits such as cardiovascular support, metabolic regulation, and neuroprotection (Saba et al., 2018). Consequently, ginseng has gained global popularity as a favored health food item (Ichim and de Boer, 2020). The slow growth rate of ginseng roots, which are the primary consumable part, necessitates a cultivation period of at least 4–6 years ginseng is particularly susceptible to soil-borne diseases (Oh et al., 1992). As a result, the preference lies in establishing ginseng fields that are initially free from concerns about soil-borne pathogens. It is noteworthy that ginseng yields have been reported to suffer substantial ranging from 30 to 60% due to root rot and soil-borne diseases (Kim et al., 2012). The primary causative agent of this root rot is *Cylindrocarpon destructans* (Park, 2001), reclassified as *Ilyonectria mors-panacis* (Cabral et al., 2012), with the Fusarium group also playing a dominant role, including species like *Fusarium solani*, *F. oxysporum*, and *F. moniliforme* (Lee, 2004).

In this study, we aim to extend the findings of Cho et al. (2023) by investigating the rhizosphere bacterial community in the monoculture of ginseng. We previously characterized this community and highlighted the role of influential bacterial families in inhibiting root rot during the early stages of ginseng monoculture also identified the primary function of these influential bacteria as nitrogen fixation. Subsequent research indicated that Pseudomonadaceae plays a crucial role in suppressing root rot after an outbreak occurs (Cho et al., 2023). Together, these previous findings suggest a dynamic progression in the establishment of a specific suppression soil, triggered by Pseudomonadaceae, following a root rot outbreak, which aligns with prior research (Yang et al., 2011). To further validate these observations, our study bridges the gap between pot experiments and field conditions. We purified and compared partial 16S rRNA library raw data from the rhizosphere of two to six-year-old ginseng plants cultivated in the field with data obtained from the pot rhizosphere in the previous studies, all under uniform computational conditions.

## Materials and methods

### Ginseng serial cultivation and nitrogen treatments

Pebbles (10 cm in size) were placed at the bottom of each pot, which measured 15 cm in diameter and 20 cm in height. Approximately 1 kg of soil was then added. The soil mixture comprised 50% autoclaved sand, 40% autoclaved ginseng field soil, and 10% raw ginseng field soil to introduce native microbiota. The raw ginseng field soil had been used for ginseng monoculture for 9 years. Each pot contained one-year-old ginseng seedlings and was treated with 50 mM of different nitrogen sources [NH_4_Cl, glutamate (Glu), aspartate (Asp), asparagine (Asn), and valine (Val)] and untreated control (distilled water) on the 0th and 10th days. The ginseng seedlings were grown under controlled light conditions (25°C for 16 h) and dark conditions (20°C for 8 h) for 20 days per cycle and each cycle had three biological replicates (*n* = 25). This study was designed to explore the potential implications of microbiota shifts in the field, particularly concerning their association with nitrogen sources and the occurrence of root rot disease.

### Rhizosphere soil collection and DNA extraction

Ginseng plants were selected for sampling in the field located at coordinates 36°56′31.7″ N and 127°45′04.8″ E, under the administration of the National Institute of Horticultural & Herbal Science in Korea. The selected ginseng plants ranged in age from 2 to 6 years since their initial planting in the field. The ginseng roots were first gently freed from bulk soil to collect rhizosphere soil. Subsequently, each ginseng root was placed in a sterile beaker containing 200 mL of pre-chilled phosphate-buffered saline (PBS) buffer. The PBS buffer was prepared by dissolving 200 mg of KCl, 8 g of NaCl, 245 mg of KH_2_PO_4_, and 1.44 g of Na_2_HPO_4_ in L of distilled water, resulting in a pH of 7.4. The beaker, containing the ginseng root in PBS, was then subjected to a sonication bath (Bandelin Electronic GmbH & Co. KG, Berlin, Germany). The rhizosphere soil, which detached from the root due to ultrasound treatment, was subsequently separated and collected through centrifugation at 3,000 × *g* for 20 min using a 1736R centrifuge (LaboGene, Seoul, Republic of Korea).

Metagenomic DNA from the rhizosphere was extracted using the FastDNA™ SPIN Kit for Soil (MP Biomedicals, Solon, Ohio, United States) according to the manufacturer’s protocol. Initially, 978 μL of sodium phosphate buffer and 122 μL of MT buffer were sequentially added to a Lysing Matrix E tube. Subsequently, the tube was subjected to homogenization using the FastPrep-24™ Classic Instrument (MP Biomedicals, Santa Ana, California, United States) set to a speed of 6.0 for 40 s. Following homogenization, the tube was centrifuged at 18,000 × *g* for 10 min. The resulting clear supernatant obtained after centrifugation was carefully transferred to a clean 2 mL tube, to which 250 μL of protein precipitation solution was added and gently inverted 10 times. After the precipitation of proteins and their subsequent removal by centrifugation, the supernatant was transferred to a 15 mL conical tube. The binding matrix settled at the bottom of the solution was resuspended, and 1 mL of this resuspended solution was added to the conical tube. The tube was then inverted for 2 min, followed by a 3 min period of settling to allow the binding matrix to collect. The top 500 μL of clear supernatant was removed without disturbing the matrix, and the matrix was resuspended by pipetting and subsequently collected through SPIN™ and centrifugation (18,000 × *g* for 10 min). The collected matrix was washed with ethanol, and the extracted DNA was eluted using 50 μL of DNase-free water.

### 16S rRNA V4 library sequencing

We conducted 16S rRNA V4 library sequencing using the Illumina adapter-linked primers 515F (5’-TCGTCGGCAGCGTCAGATGTGTATAAGAGACAG GTGCCAGCMGCCGCGGTAA-3′) and 805R (5’-GTCTCGTGGGCTCGGAGATGTGTATA AGAGACAGGACTACHVGGGTATCTAATCC-3′). This sequencing was performed with metagenomic DNA extracted from the rhizosphere of ginseng cultivated in a field setting. The KAPA HiFi HotStart ReadyMix (Kapa Biosystems, Wilmington, Massachusetts, United States) was utilized for polymerase chain reaction (PCR) amplification. The PCR conditions employed were as follows: an initial denaturation step at 95°C for 3 min, followed by 25 cycles consisting of denaturation at 95°C for 30 s, annealing at 55°C for 30 s, and extension at 72°C for 30 s. A final extension step was carried out at 72°C for 5 min. Subsequently, the sequencing process was outsourced to Macrogen (Seoul, Republic of Korea). To attach linker and barcode sequences, a second PCR thermal cycling step was conducted with the following conditions: an initial denaturation at 95°C for 3 min, followed by 8 cycles consisting of denaturation at 95°C for 30 s, annealing at 55°C for 30 s, and extension at 72°C for 30 s. A final extension step was carried out at 72°C for 5 min. The obtained sequences were read on a MiSeq 2 × 300 bp platform (Illumina, San Diego, California, United States) to facilitate downstream analysis.

### Metagenome data processing and ASV clustering

Metagenome data underwent rigorous processing procedures using a high-performance workstation equipped with an AMD Ryzen Threadripper 3,970X processor and 64 GiB of DDR4 RAM, running Ubuntu 18.04. The raw data, including previously processed datasets and newly acquired data from field-cultivated ginseng samples, were integrated to optimize error rate prediction through the divisive amplicon denoising algorithm (DADA) (Callahan et al., 2016) and minimize fragmentation. For the ginseng serial cultivation experiment, involving 40 pot samples, and the nitrogen source treatment, involving 30 samples, as well as the field study comprising 15 samples, the 805R Illumina adapter (5′-GTCTCGTGGGCTCGGAGATGTGTATAAGAGACAGGGACTACHVHHHTWTCTAAT-3′) was employed. Primer sequences were meticulously removed using Cutadapt (version 2.10; Martin, 2011). The resultant primer-free raw sequences, obtained from the MiSeq platform with a 2 × 300 bp configuration, were subsequently imported into R (version 4.0.4). Sequences with an average accuracy of less than 99.9% at the 3′ end were trimmed. The DADA2 package (version 1.16.0) in R was employed to model the error rate for the trimmed sequences. Forward and reverse reading sequences were merged following sequence modification by DADA. Initially, clustering was performed based on perfectly identical sequences. Chimeric and non-bacterial amplicon sequence variant (ASV), classified using IDTAXA (Murali et al., 2018) referencing SILVA 138 SSU during the first clustering step, were removed to enhance alignment accuracy and to eliminate an additional 2 bp introduced by the 515F-806R amplicon. Following the removal of the 2 bp, a second clustering step based on completely identical sequences was executed. The ASVs generated during the second clustering were subjected to re-identification using the IDTAXA approach. The results of these procedures, including the number of reads, are presented in Supplementary Table S1.

### Bacterial community analysis

Bacterial community analysis was conducted using a combination of software tools in the R programming environment. The analysis utilized phyloseq (version 1.32.0; McMurdie and Holmes, 2013), vegan (version 2.5–0; Oksanen et al., 2012), and DESeq2 (version 1.18.2; Love et al., 2014). Additional details and code for this analysis are available in the associated GitHub repository^1^^,^ ^2^^,^ ^3^.

### Pathway prediction

For predicting the pathways of each sample, we employed PICRUSt2 (Phylogenetic Investigation of Communities by Reconstruction of Unobserved States; version 2.3.0-b) with KEGG and MetaCyc databases. This analysis was performed in Python (version 3.7.8) (Douglas et al., 2020).

### Beta diversity modification

To account for variations in α diversity between field and pot samples, we utilized the β-nearest taxon index (βNTI) (Stegen et al., 2012). The maximum likelihood tree required for βNTI calculations was generated using RAxML-NG (Randomized Axelerated Maximum Likelihood Next Generation, version 1.0.2) (Kozlov et al., 2019).

### Code repository

The R code used for these analyses is available in the following GitHub repository: https://github.com/gyeongjunCho/R-code-of-Ph.D.-Thesis (Cho, 2023).

## Results and discussion

### α diversity of ginseng rhizosphere microbial communities

**Figure 1:**
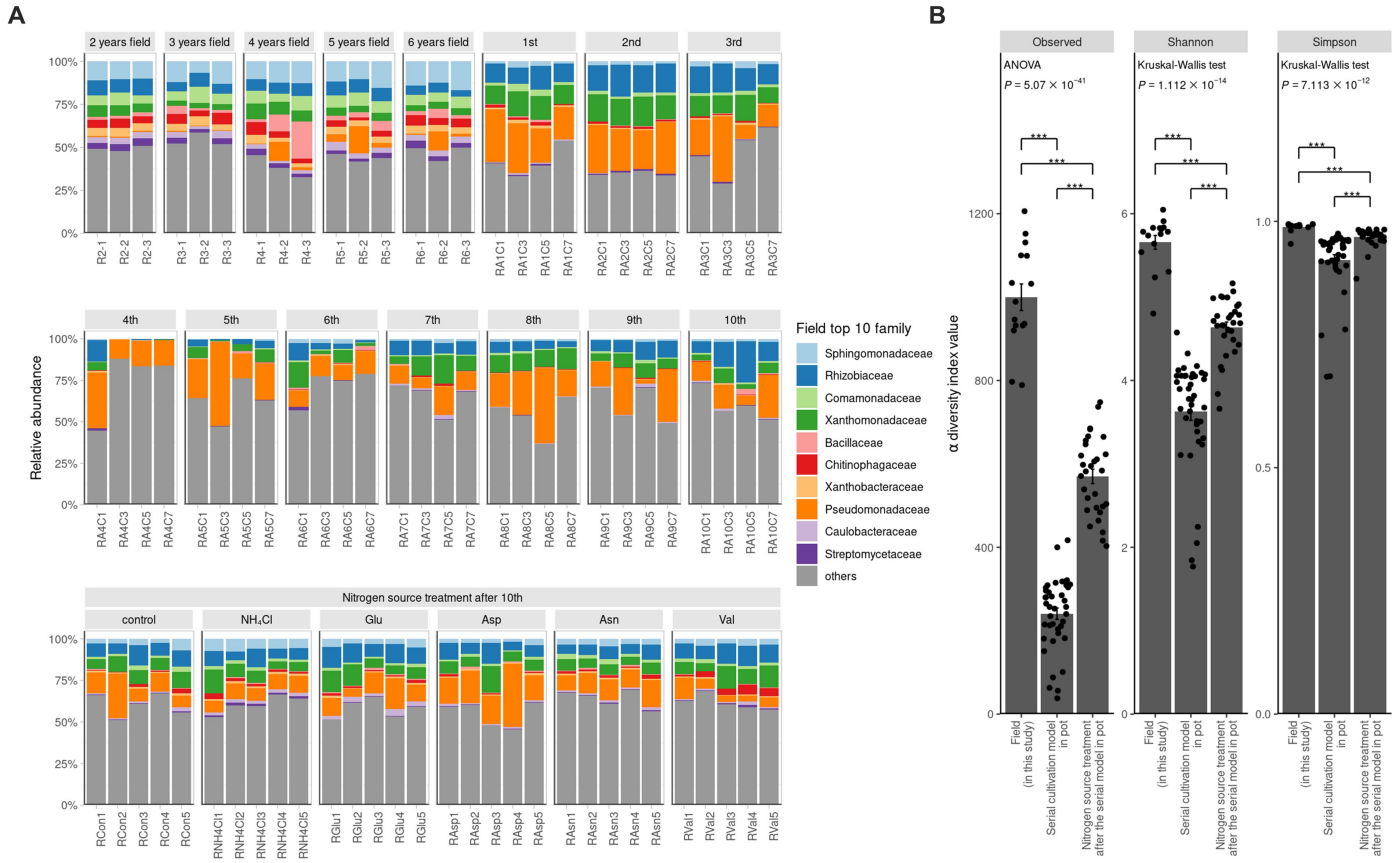
Ginseng rhizosphere bacterial community composition. **(A)** Relative abundance is portrayed by bar graph at family levels. The colored families are selected in order from the top 1 to 10 in the average value of relative abundance in the field. *α* diversity of field, contiuous monoculture, and nitrogen source treatment sample. **(B)** Alpha diversity is compared among the two previous study and this field study. The observed value means the number of ASVs. Shannon index and Simpson index refer to richness and evenness of *α* diversity. All three indicators of *α* diversity is significantly different, of which the field samples were the highest and the serial cultivation samples were the lowest. If the indicator is of normal distribution and equal variance, ANOVA is performed. Otherwise, Kruskal–Wallis test is performed. Their *post hoc* test is Tukey’s HSD (*P ≦ 0.05, **P ≦ 0.01, ***P ≦ 0.001).

The rarefaction curve demonstrates the effectiveness of ASV clustering, revealing a minimal likelihood of encountering new ASVs with increasing read depth (Supplementary Figure S1). Furthermore, we observed significant variations in diversity among different samples, including field samples, rhizosphere samples from serial cultivation in pots, and rhizosphere samples subjected to nitrogen source treatments. These differences were evident in the ASV counts, Shannon index, and Simpson index (Figure 1B). Notably, the α diversity indices followed a consistent order, with field samples exhibiting the highest diversity, followed by nitrogen treatment samples in pots and serial cultivation samples in pots. In the context of continuous ginseng replanting and harvesting in pots, the initial influencers displayed an initial suppression of root rot progression. However, their relative abundance in the rhizosphere gradually decreased, eventually leading to a peak in disease progression (Cho et al., 2023). When different nitrogen sources were applied to pots containing bulk soil from previous replanting pots, a significant increase in Pseudomonadaceae was observed. Interestingly, the disease was effectively suppressed in Asp (aspartate) treatment but significantly increased in Val (valine) treatment (Cho, 2023). This transition in the rhizosphere community dynamics during continuous ginseng cultivation in pots highlighted the transfer of disease suppression responsibility from the initial community influencers to Pseudomonadaceae. Consequently, distinct levels of disease occurrence were observed across these periods (Cho, 2023).

### Microbial communities β diversity

**Figure 2:**
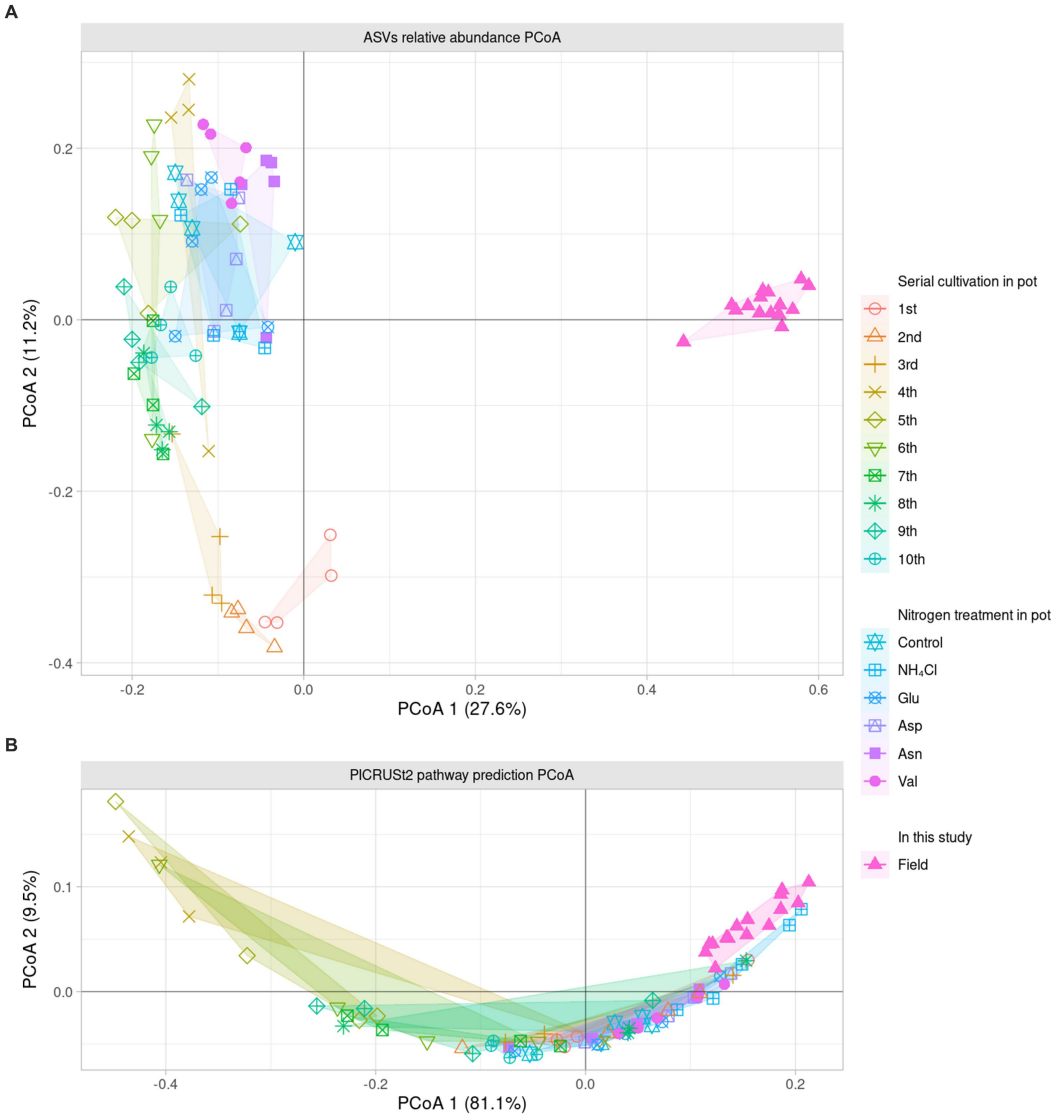
Dissimilarity analysis of bacterial community and their predicted metabolic pathway The PCoA displays the Bray–Curtis distance calculated as the relative abundance of **(A)** ASVs and **(B)** pathway prediction of PICRUSt2 in two dimensions. Each dot indicates a sample, and the shape and color of the dots represent the condition of each sample. The number in parentheses in the axis title presents that the proportion of variation explained by each axis. The field samples are the most similar with NH_4_Cl treatment in the metabolic prediction.

To compare the field and the pot rhizosphere with β diversity, relative abundance was calculated (Figure 1A) and principal coordinates analysis (PCoA) with Bray-Curtis distance was performed at the ASV level (Figure 2A). The result indicated that there was a significant difference between the pots and fields, as well as that pots could not describe fields’ results. However, Bray-Curtis distance from PCoA calculated with PICRUSt2 pathway prediction showed high similarity between field and NH_4_Cl treated pot samples (Figure 2B). As a result of analysis with permutational multivariate analysis of variance (PERMANOVA) with Bray–Curtis distance, the field and almost all pot samples were significantly different, but there was no significantly difference between field and NH_4_Cl treatments (Figure 2B and Supplementary Table S2). The distribution between the nitrogen source treated rhizosphere and the late replanting rhizosphere overlapped the relationship because the design of these previous studies at pot was well explained (Cho, 2023). These results indicate that the composition and diversity of rhizosphere microbiota communities are significantly affected by different nitrogen sources. The α diversity of the field was significantly higher than other treatments, and field the β diversity in PCoA at the ASVs level was different. Additionally, PCoA indicated that the nitrogen source treatment study was the directly continuous study of the cycling study because the nitrogen source treatment experiment was generally far from the first to third cycling samples in the ginseng cycling and overlapped with the 4th to 10th cycling samples (Figure 2A).

**Figure 3:**
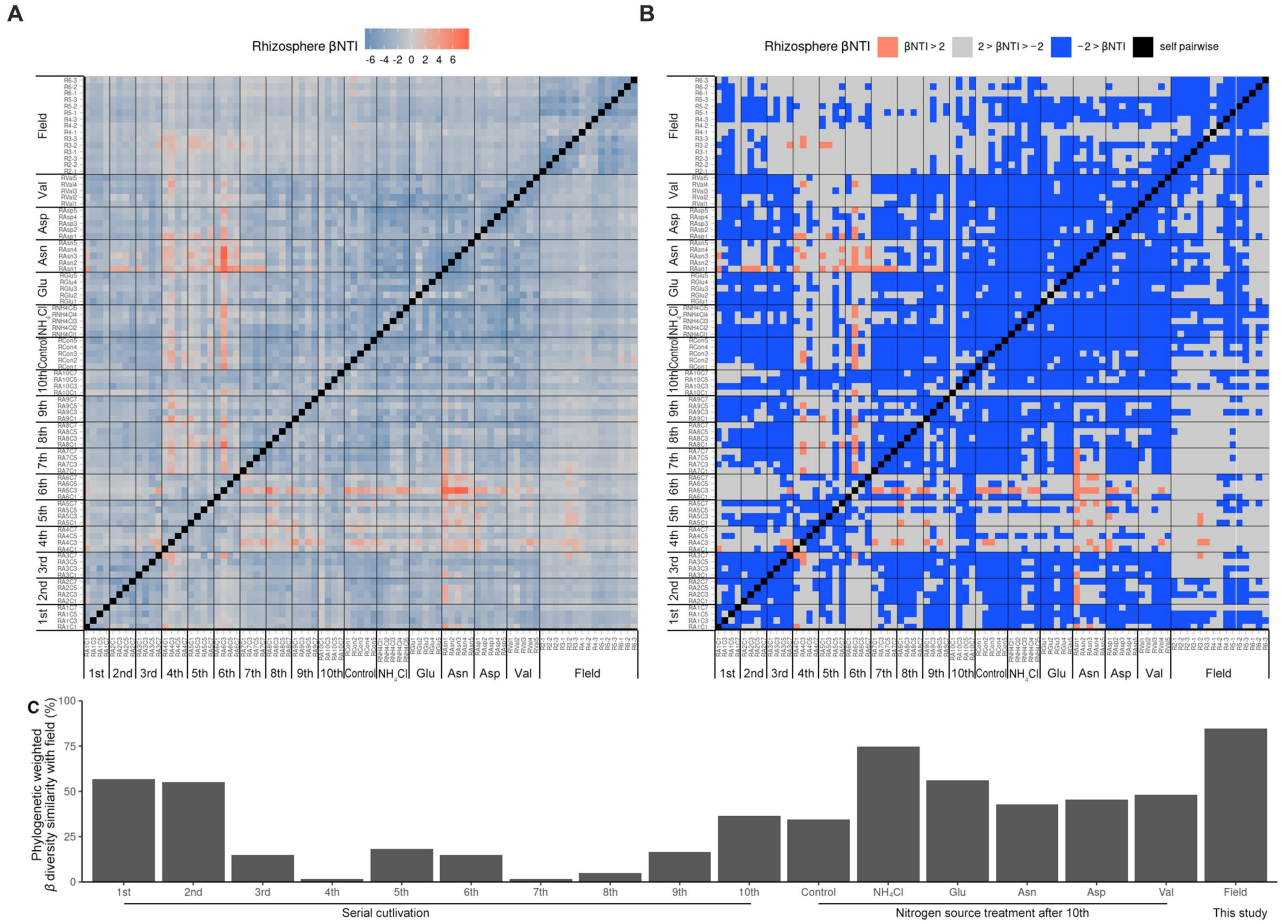
Phylogenetic weighted *β* diversity analysis. *β* NTI means the distance of phylogenetic *β* diversity of a pair of samples. Significantly, *β* NTI < −2 indicated that a pair of communities phylogenetically close and *β* NTI > 2 indicated that a pair of community communities phylogenetically far. Heatmap of *β* NTI **(A)** is simplified by *β* NTI < −2 to blue, *β* NTI > 2 to pinky red **(B)**. Phylogenetic weighted *β* diversity similarity with field **(C)** is calculated by the phylogenetically close (*β*NTI < −2) ratio compared with field result.

Since it was difficult to compare the field with the ASV level alone, the phylogenetic tree of the 16S rRNA V4 region of ASV was calculated as maximum likelihood, and beta NTI was calculated together with the phylogenetic tree and the relative abundance (Figures 3A,B). Importantly, the similarity of microbiota community structure between the field and pot rhizospheres was very high (βNTI < −2). When measuring how similar the phylogenetic diversity (β diversity) was, it was found to be the highest at 84.79% when compared to the field itself, and the second highest at 74.67% when compared to the field treated with NH_4_Cl treatment (Figure 3C). The treatment first cycle, Glu treatment, and the second cycle had the highest similarity to field treatment (56.67, 56.00, and 55.00%, respectively). The remaining similarity did not exceed 50%. Assuming that the difference in β diversity is primarily influenced by the difference in α diversity, it is expected that the difference will decrease when considering β diversity with the phylogenetic tree. Therefore, βNTI was calculated.

Therefore, it is reasonable to group the field rhizospheres and the NH_4_Cl-treated rhizospheres when comparing the relative abundance. Since the phylogenetic beta diversity was similar between the field rhizosphere and the NH_4_Cl-treated rhizosphere, it was decided that relative abundance could be compared in the microbial community.

### Bacteria sharing between community

**Figure 4:**
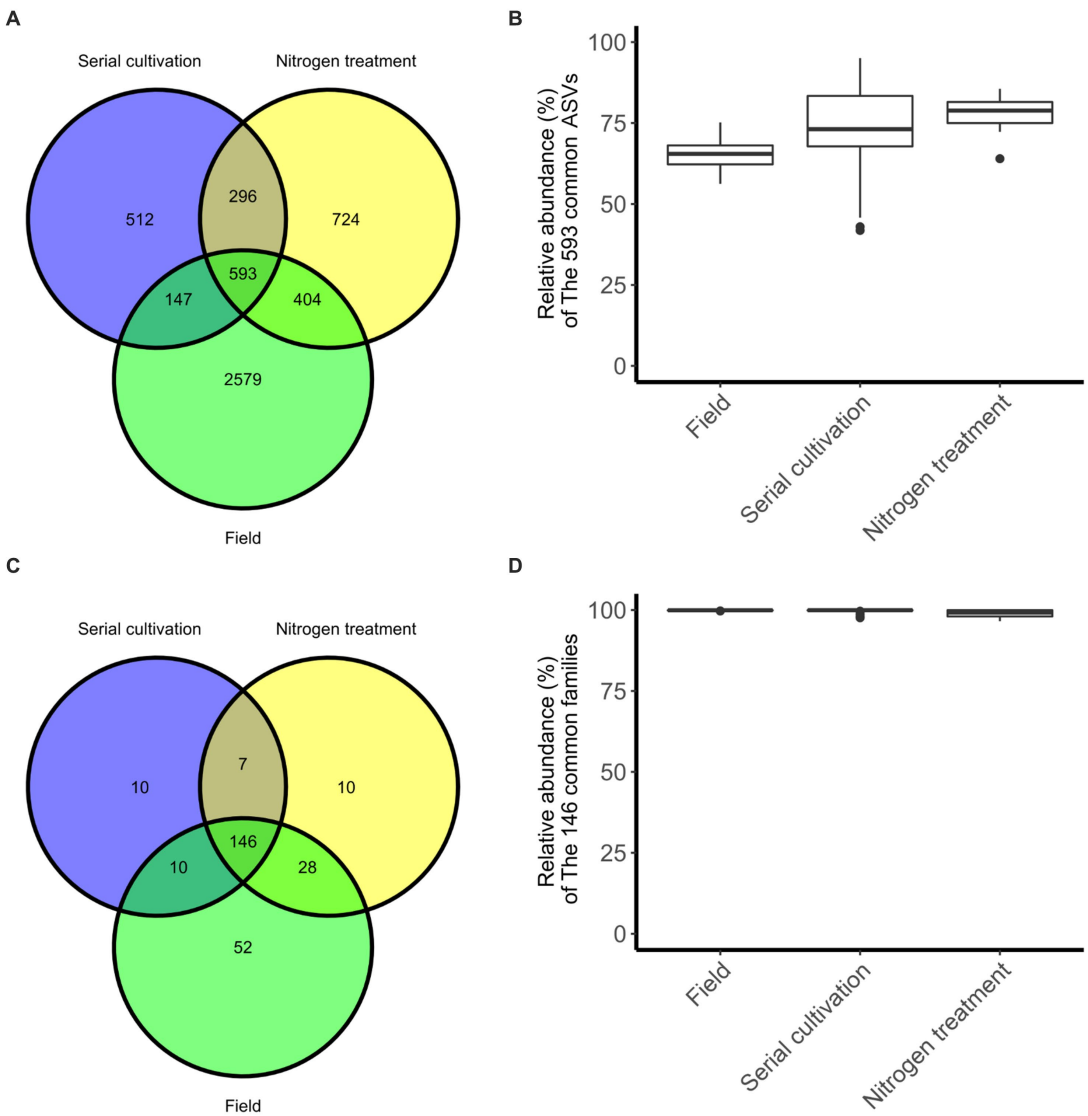
The degree of sharing at the differential phylogenetic level. Sharing of ASV **(A)** and family **(C)** levels are represented in Venn diagram. The relative abundance of bacteria, 593 ASVs **(B)** and 146 families **(D)**, belonging to the three groups is displayed by the box-whisker diagram.

In our investigation, we quantified and visualized the shared microbial taxa using a Venn diagram (Figure 4A). We identified 3,723 ASVs in the field rhizosphere, 1,548 ASVs in the rhizosphere of the serial cultivation study, and 2017 ASVs in the rhizosphere of the nitrogen source treatment. Remarkably, 593 ASVs were found to be shared among all three categories, with their relative abundance medians measuring 65% in the field, 73% in the serial cultivation, and 78% in the nitrogen source treatment (Figure 4B). At the family level, we observed 236 ASVs in the field, 173 in the serial cultivation, and 192 in the nitrogen source treatment (Figure 4C). The relative abundance median of the 146 shared families was consistently high, with values of 99% in the field, 99% in the serial cultivation, and 99% in the nitrogen source treatment (Figure 4D). This indicated that family-level abundance comparisons were valid. To offer an alternative approach for comparison, we considered the predicted pathways using PICRUSt2, as the functional genes of the bacteria were expected to exhibit limited variation across different bacterial taxa. Our PCoA of the predicted pathways revealed significant distinctions in the bacterial community composition of the field rhizosphere compared to the pot rhizosphere, except in the case of the NH_4_Cl treatment (Figure 2B; Table 1; Supplementary Table S2). The analysis of phylogenetic β diversity and the PCoA results of the predicted pathways provided further evidence that the bacterial community characteristics in the field closely resembled those of the NH_4_Cl treatment.

**Table 1:** Pairwise PERMANOVA result compared to rhizosphere metabolism prediction in ginseng field.

Compare	Sum of squares	*F* model	*R*^2^	*P*	*P_adj_*
Control vs. Field	0.267	39.897	0.689	0.001	0.010*
Asn vs. Field	0.176	17.923	0.499	0.001	0.010*
Asp vs. Field	0.144	18.735	0.51	0.001	0.010*
Glu vs. Field	0.194	20.373	0.531	0.001	0.010*
Val vs. Field	0.138	22.13	0.551	0.001	0.010*
NH_4_Cl vs. Field	0.013	1.897	0.095	0.168	0.253*
1st vs. Field	0.185	21.681	0.561	0.003	0.027*
2nd vs. Field	0.139	13.697	0.446	0.001	0.010*
3rd vs. Field	0.225	24.283	0.588	0.002	0.019*
4th vs. Field	0.475	29.329	0.633	0.001	0.010*
5th vs. Field	0.573	61.698	0.784	0.001	0.010*
6th vs. Field	0.423	29.982	0.638	0.001	0.010*
7th vs. Field	0.382	36.198	0.68	0.001	0.010*
8th vs. Field	0.12	8.468	0.332	0.001	0.010*
9th vs. Field	0.304	23	0.575	0.001	0.010*
10th vs. Field	0.407	83.237	0.83	0.001	0.010*

*p*-value of PERMANOVA is 0.001. The PERMANOVA and pairwise PERMANOVA are calculated Bray–Curtis dissimilarity of PICRUSt2 metabolic pathway prediction. The *P*_adj_ is calculated by false discovery rate method. *P_adj_ < 0.05.

### Characteristics of field rhizosphere

**Figure 5:**
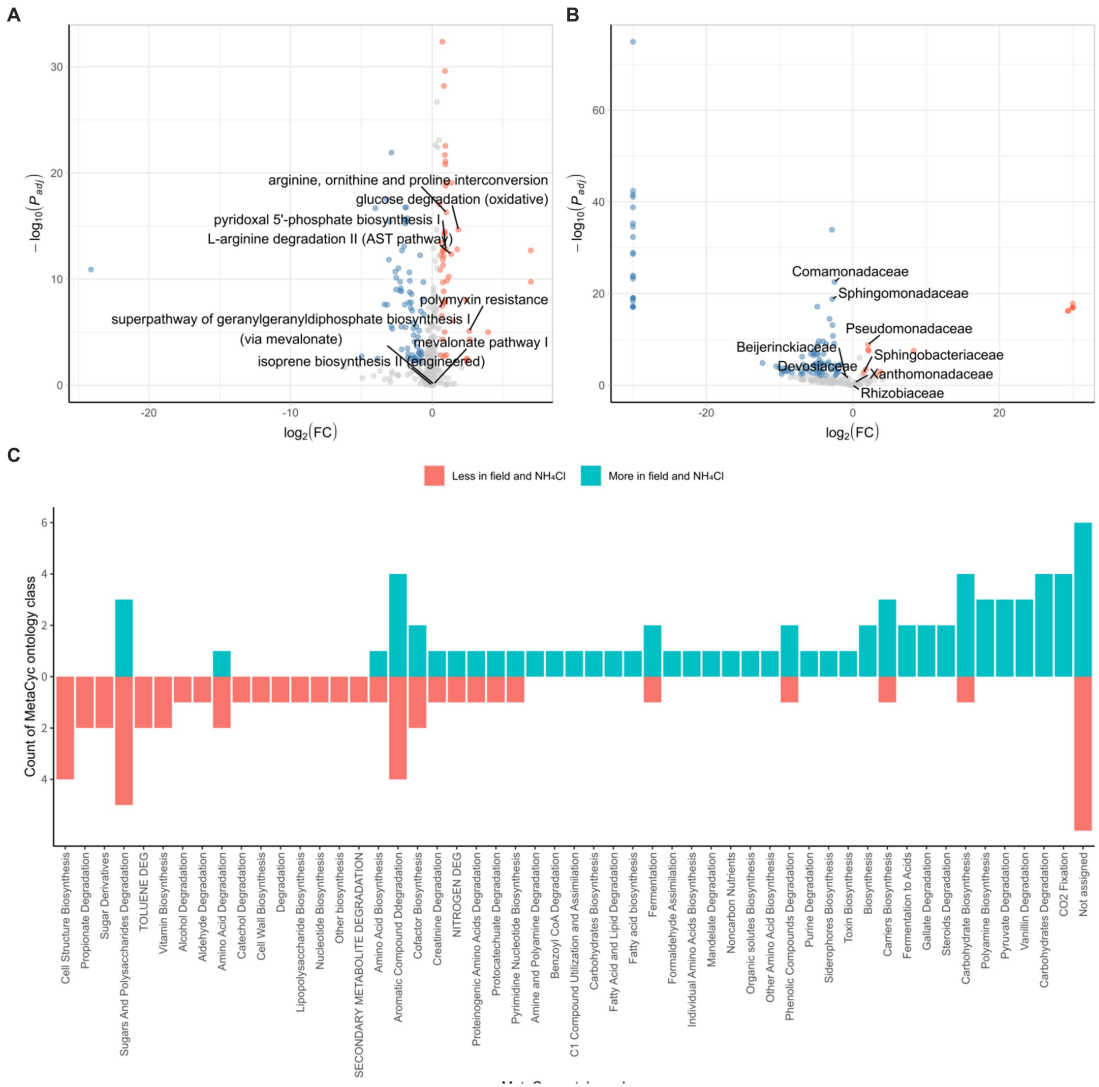
Characterization of the bacterial community in the field and NH_4_Cl treatment. DESeq2 analyses are conducted by PICRUSt2 predicted pathway using MetaCyc database **(A)** and relative family abundance **(B)**. *P_adj_* is calculated by the FDR method. Red dots indicated by log_2_ (FC) > 0.5 and *P_adj_* < 0.01 are less pathway or family in field and NH_4_Cl treatment. Blue dots displayed by log_2_ (FC) < −0.5 and *P_adj_* < 0.01 are more pathways or families in the field and NH_4_Cl. In field and NH_4_Cl treatment, ‘Arginine, ornithin and proline interconversion,’ ‘glucose degradation (oxidative),’ ‘l-arginine degradation II (AST pathway),’ ‘pyridoxal 5’-phosphate biosynthesis I’, and ‘polymyxin resistance’ which had been negative correlation against root rot progression in the previous study, are less significant. ‘isoprene biosynthesis II (engineered),’ ‘mevalonate pathway I,’ and ‘super pathway of geranylgeranyl diphosphate biosynthesis (via mevalonate)’ which had been negative correlation against *F. solani* density are not differed significantly. **(C)** MetaCyc ontology analyses are performed by counting that significantly different pathways are classified the ontology group provided by MetaCyc (https://metacyc.org/). (FC, fold change).

We conducted a comparative analysis between the field and NH_4_Cl treatment group and another group comprising various rhizosphere samples using the DESeq2 R package. In the field and NH_4_Cl treatment groups, we observed a significant reduction in the abundance of pathways that had previously shown negative correlations with root rot disease progression in a study involving different nitrogen sources (Wang et al., 2019; Ding et al., 2021). These pathways included ‘arginine, ornithine, and proline interconversion,’ ‘glucose degradation (oxidative),’ ‘pyridoxal 5’-phosphate biosynthesis I,’ ‘l-arginine degradation II (AST pathway),’ and ‘polymyxin resistance’ (Figure 5A). In contrast, the ‘terpenoid biosynthesis’ pathway, including the ‘super pathway of geranylgeranyl diphosphate biosynthesis I (via mevalonate),’ ‘mevalonate pathway I,’ and ‘isoprene biosynthesis II (engineered),’ which had shown negative correlations with *F. solani* population density in the previous nitrogen source treatment study (Griffin, 1970; Morgan and Timmer, 1984), remained unchanged. All of the identified pathways were subjected to ontology analysis at the MetaCyc ontology class level (Figure 5C), revealing notable differences in the carbon and nitrogen source circulation within the rhizosphere bacterial community between the field group and the other samples.

We further explored the DESeq2 results at family level, focusing on families that were instrumental in influencing the initial rhizosphere community to inhibit root rot progression. In the context of the serial cultivation study, these influential families included Rhizobiaceae, Beijerinckiaceae, Devosiaceae, Xanthomonadaceae, Sphingobacteriaceae, Sphingomonadaceae, and Comamonadaceae. Additionally, Pseudomonadaceae, which had been associated with pathways negatively correlated with root rot progression and *F. solani* population density in the nitrogen source treatment (Figure 5B). Within this analysis, we found that Comamonadaceae and Sphingomonadaceae were more abundant in the field and NH_4_Cl treatment group, whereas Pseudomonadaceae and Sphingobacteriaceae were less abundant in this group. These findings were consistent with the results from the field where ginseng was grown for 2–6 years. The pathways negatively correlated with root rot progression, including ‘arginine, ornithine, and proline interconversion,’ ‘glucose degradation (oxidative),’ ‘pyridoxal 5’-phosphate biosynthesis I,’ ‘l-arginine degradation II (AST pathway),’ and ‘polymyxin resistance,’ were less abundant in the Field and NH4Cl treatment group. Notably, the density of *F. solani*, the root rot causative pathogen, which had shown negative correlations with pathways in previous studies, did not differ between the field and NH_4_Cl treatment group and the other samples (Morgan and Timmer, 1984). Regarding the initial community influencers identified in the continuous study, we observed varying levels of abundance in the field and NH_4_Cl treatment groups. However, Pseudomonadaceae, which had played a significant role in the eight identified pathways, was significantly less abundant in the field and NH_4_Cl treatment group. This observation suggests that the rhizosphere field may be more susceptible to disease, independent of *F. solani* population density, and emphasizes the potential for disease suppression by Pseudomonadaceae due to its lower abundance in the field compared to other samples.

A previous field study extends and compares the findings from previously reported artificial continuous cultivation of ginseng and nitrogen source studies (Cho, 2023), both of which are associated with the rhizosphere community. This investigation focuses on the later stages of the continuous cultivation, providing an evaluation of the ginseng rhizosphere in a field setting. Our results demonstrate that the initial community in the field deteriorated within 2 years after planting and closely resembled the NH_4_Cl treatment group at the time of sampling. This shift is attributed to the predominant use of fertilizers primarily composed of ammonium rather than amino acids during continuous cultivation. Furthermore, we observed a reduction in disease-correlated pathways and Pseudomonadaceae, which had played a crucial role in these pathways in the previous study, in the field of rhizosphere. This study underscores the susceptibility of the field rhizosphere to disease, corroborating previous research findings. Considering our findings, we propose the potential for root rot disease suppression in ginseng fields using Asp and Pseudomonadaceae.

## Data availability statement

The original contributions presented in the study are included in the article/Supplementary material. The project has been deposited to the NCBI repository under the BioProject accession numbers PRJNA971581, PRJNA971514, and PRJNA1126992.

## Author contributions

GC: Writing – original draft, Data curation, Formal analysis, Investigation, Methodology, Software, Validation, Visualization. D-RK: Data curation, Formal analysis, Investigation, Methodology, Software, Validation, Visualization, Writing – original draft. Y-SK: Writing – original draft, Conceptualization, Funding acquisition, Project administration, Resources, Supervision, Writing – review & editing.

## Footnotes

1
https://github.com/joey711/phyloseq

2
https://github.com/vegandevs/vegan

3
https://github.com/mikelove/DESeq2

## References

[ref1] Baeg I.-H. So S.-H. (2013). The world ginseng market and the ginseng (Korea). J. Ginseng Res. 37, 1–7. doi: 10.5142/jgr.2013.37.1, PMID: 23717152 PMC3659626

[ref2] Berg G. Smalla K. (2009). Plant species and soil type cooperatively shape the structure and function of microbial communities in the rhizosphere. FEMS Microbiol. Ecol. 68, 1–13. doi: 10.1111/j.1574-6941.2009.00654.x, PMID: 19243436

[ref3] Cabral A. Groenewald J. Z. Rego C. Oliveira H. Crous P. W. (2012). Cylindrocarpon root rot: multi-gene analysis reveals novel species within the *Ilyonectria radicicola* species complex. Mycol. Prog. 11, 655–688. doi: 10.1007/s11557-011-0777-7

[ref4] Callahan B. J. McMurdie P. J. Rosen M. J. Han A. W. Johnson A. J. A. Holmes S. P. (2016). DADA2: high-resolution sample inference from Illumina amplicon data. Nat. Methods 13, 581–583. doi: 10.1038/nmeth.3869, PMID: 27214047 PMC4927377

[ref5] Cho G. (2023). PhD dissertation. Republic of Korea: Gyeongsang National University.

[ref6] Cho G. Kim D.-R. Kwak Y.-S. (2023). Transition from ginseng root rot disease-conducive soil to suppressive soil meditated by Pseudomonadaceae. Microbiol. Spectr. 11:e0115023. doi: 10.1128/spectrum.01150-23, PMID: 37404179 PMC10433981

[ref7] Cook R. (2014). “Plant health management: pathogen suppressive soils” in Encyclopedia of agriculture and food systems. Amsterdam, Netherlands: Elsevier. doi: 10.1016/0038-0717(76)90056-0

[ref8] Ding S. Shao X. Li J. Ahammed G. J. Yao Y. Ding H.. (2021). Nitrogen forms and metabolism affect plant defence to foliar and root pathogens in tomato. Plant Cell Environ. 44, 1596–1610. doi: 10.1111/pce.14019 33547690

[ref9] Douglas G. M. Maffei V. J. Zaneveld J. R. Yurgel S. N. Brown J. R. Taylor C. M.. (2020). PICRUSt2 for prediction of metagenome functions. Nat. Biotechnol. 38, 685–688. doi: 10.1038/s41587-020-0548-6 32483366 PMC7365738

[ref10] Griffin G. J. (1970). Carbon and nitrogen requirements for macroconidial germination of *Fusarium solani*: dependence of conidial density. Can. J. Microbiol. 16, 733–740. doi: 10.1139/m70-125, PMID: 5484061

[ref11] Guo Q. Li Y. Lou Y. Shi M. Jiang Y. Zhou J.. (2019). *Bacillus amyloliquefaciens* Ba13 induces plant systemic resistance and improves rhizosphere microecology against tomato yellow leaf curl virus disease. Appl. Soil Ecol. 137, 154–166. doi: 10.1016/j.apsoil.2019.01.015

[ref12] Hiltner L. (1904). Uber nevere erfahrungen und probleme auf dem gebiet der boden bakteriologie und unter besonderer beurchsichtigung der grundungung und broche. Arbeit Deut Landw Ges. Berlin 98, 59–78.

[ref13] Ichim M. C. de Boer H. J. (2020). A review of authenticity and authentication of commercial ginseng herbal medicines and food supplements. Front. Pharmacol. 11:2185. doi: 10.3389/fphar.2020.612071 PMC7832030 33505315

[ref14] Kim Y.-S. Lee M.-S. Yeom J.-H. Song J.-G. Lee I.-K. Yeo W.-H.. (2012). Screening of antagonistic bacteria for biological control of ginseng root rot. Kor. J. Mycol. 40, 44–48. doi: 10.4489/KJM.2012.40.1.044

[ref15] Korean Culture and Information Service (2014). Guide to Korean culture. Republic of Korea: Ministry of Culture, Sports and Tourism.

[ref16] Kozlov A. M. Darriba D. Flouri T. Morel B. Stamatakis A. (2019). RAxML-NG: a fast, scalable and user-friendly tool for maximum likelihood phylogenetic inference. Bioinformatics 35, 4453–4455. doi: 10.1093/bioinformatics/btz305, PMID: 31070718 PMC6821337

[ref17] Lee S.-G. (2004). *Fusarium* species associated with ginseng (*Panax Ginseng*) and their role in the root-rot of ginseng plant. Res. Plant Dis. 10, 248–259. doi: 10.5423/RPD.2004.10.4.248

[ref18] Love M. I. Huber W. Anders S. (2014). Moderated estimation of fold change and dispersion for RNA-seq data with DESeq2. Genome Biol. 15:550. doi: 10.1186/s13059-014-0550-8, PMID: 25516281 PMC4302049

[ref19] Martin M. (2011). Cutadapt removes adapter sequences from high-throughput sequencing reads. EMBnet J. 17, 10–12. doi: 10.14806/ej.17.1.200

[ref20] McMurdie P. J. Holmes S. (2013). Phyloseq: an R package for reproducible interactive analysis and graphics of microbiome census data. PLoS One 8:e61217. doi: 10.1371/journal.pone.0061217, PMID: 23630581 PMC3632530

[ref21] Morgan K. T. Timmer L. W. (1984). Effect of inoculum density, nitrogen source and saprophytic fungi on Fusarium wilt on Mexican lime. Plant Soil 79, 203–210. doi: 10.1007/BF02182342

[ref22] Murali A. Bhargava A. Wright E. S. (2018). IDTAXA: a novel approach for accurate taxonomic classification of microbiome sequences. Microbiome 6, 1–14. doi: 10.1186/s40168-018-0521-5 30092815 PMC6085705

[ref23] Oh S. Yu Y. Kim K. Cho D. (1992). “Studies on control of soil-borne diseases and insects of ginseng and development of antifungal compound” in Ginseng Cultivation Bul, (Taejon, Korea: Korea Ginseng and Tobacco ResearchInst., Ginseng Cultivation Bull), 121–184.

[ref24] Oksanen J. Blanchet F. G. Kindt R. Legendre P. Minchin P. R. O'Hara R. B.. (2012). Vegan: community ecology package. Software. Available at: http://CRAN.R-project.org/package=vegan

[ref25] Park K. (2001). Fitness analysis of the forecasting model for root rot progress of ginseng based on bioassay and soil environmental factors. Res. Plant Dis. 7, 20–24.

[ref26] Pinton R. Varanini Z. Nannipieri P. (2007). The rhizosphere: biochemistry and organic substances at the soil-plant interface. Boca Raton, FL, USA: CRC press.

[ref27] Saba E. Jeong D. Irfan M. Lee Y. Y. Park S.-J. Park C.-K.. (2018). Anti-inflammatory activity of rg3-enriched Korean red ginseng extract in murine model of sepsis. Evid. Based Complement. Alternat. Med. 2018, 1–11. doi: 10.1155/2018/6874692, PMID: 30405742 PMC6201491

[ref28] Schlatter D. Kinkel L. Thomashow L. S. Weller D. Paulitz T. (2017). Disease suppressive soils: new insights from the soil microbiome. Phytopathology 107, 1284–1297. doi: 10.1094/PHYTO-03-17-0111-RVW, PMID: 28650266

[ref29] Stegen J. C. Lin X. Konopka A. E. Fredrickson J. K. (2012). Stochastic and deterministic assembly processes in subsurface microbial communities. ISME J. 6, 1653–1664. doi: 10.1038/ismej.2012.22, PMID: 22456445 PMC3498916

[ref30] Wang M. Gu Z. Wang R. Guo J. Ling N. Firbank L. G.. (2019). Plant primary metabolism regulated by nitrogen contributes to plant-pathogen interactions. Plant Cell Physiol. 60, 329–342. doi: 10.1093/pcp/pcy211, PMID: 30388252

[ref31] Weller D. M. Raaijmakers J. M. Gardener B. B. M. Thomashow L. S. (2002). Microbial populations responsible for specific soil suppressiveness to plant pathogens. Annu. Rev. Phytopathol. 40, 309–348. doi: 10.1146/annurev.phyto.40.030402.110010 12147763

[ref32] Yang M.-M. Mavrodi D. V. Mavrodi O. V. Bonsall R. F. Parejko J. A. Paulitz T. C.. (2011). Biological control of take-all by fluorescent *Pseudomonas* spp. from Chinese wheat fields. Phytopathology 101, 1481–1491. doi: 10.1094/PHYTO-04-11-0096, PMID: 22070279

