# Supplementary material for: Role of microbial communities and nitrogen sources in suppressing root rot disease during ginseng cultivation
Source: Front Microbiol. 2024 Jul 4;15:1396686. doi: 10.3389/fmicb.2024.1396686 (PMC11254850; doi:10.3389/fmicb.2024.1396686)
Supplement: Supplementary file 1 [file Presentation_1.pdf]

Supplementary Information

for

Role of Microbial Communities and Nitrogen Sources in Suppressing Root Rot Disease during Ginseng Cultivation

Gyeongjun Cho[1], Da-Ran Kim, and Youn-Sig Kwak*

This file includes 2 tables and 1 figure.

Table S1. Miseq raw reads quality control results

Sample	Raw forward	Raw reverse	Filtered forward	Filtered reverse	Denoised forward	Denoised reverse	Merged reads	Removed chimera	Only bacteria
RCon1	125,360	125,360	107,838	107,838	109,609	109,819	106,705	99,412	67,726
RCon2	122,819	122,819	116,658	116,658	99,120	99,526	96,510	93,081	70,382
RCon3	129,538	129,538	114,752	114,752	96,239	96,487	93,997	90,938	65,536
RCon4	124,641	124,641	109,777	109,777	105,478	105,789	102,677	96,782	60,837
RCon5	156,541	156,541	111,941	111,941	96,460	96,603	93,771	91,855	72,352
RNH_4Cl1	128,563	128,563	103,365	103,365	105,831	106,145	103,256	101,577	80,563
RNH_4Cl2	128,269	128,269	99,683	99,683	97,867	98,229	95,718	94,391	65,036
RNH_4Cl3	116,033	116,033	107,217	107,217	116,219	116,422	113,086	109,394	80,193
RNH_4Cl4	130,477	130,477	88,573	88,573	103,637	103,979	100,407	97,653	71,379
RNH_4Cl5	104,770	104,770	110,152	110,152	78,086	78,162	75,932	75,075	55,009
RGlu1	114,986	114,986	111,220	111,220	96,866	97,260	92,682	86,192	67,704
RGlu2	129,378	129,378	100,664	100,664	101,367	101,611	99,298	97,276	84,326
RGlu3	122,435	122,435	97,551	97,551	99,420	99,565	95,943	91,505	68,724
RGlu4	139,571	139,571	106,885	106,885	101,121	101,369	98,253	95,483	72,898
RGlu5	116,129	116,129	98,141	98,141	107,814	108,116	104,239	100,006	78,999

**Sample**	**Raw forward**	**Raw reverse**	**Filtered forward**	**Filtered reverse**	**Denoised forward**	**Denoised reverse**	**Merged reads**	**Removed chimera**	**Only bacteria**
RAsp1	129,665	129,665	99,103	99,103	102,172	102,266	99,719	96,874	82,492
RAsp2	136,800	136,800	102,553	102,553	98,334	98,455	95,473	91,999	55,706
RAsp3	111,586	111,586	100,753	100,753	105,663	106,045	102,725	97,325	66,435
RAsp4	123,821	123,821	102,609	102,609	87,425	87,631	85,526	83,366	61,473
RAsp5	110,120	110,120	109,616	109,616	108,475	108,768	104,993	101,627	74,863
RAsn1	130,438	130,438	107,453	107,453	105,960	106,250	102,847	98,677	75,645
RAsn2	117,561	117,561	99,486	99,486	114,920	115,279	111,923	107,084	74,635
RAsn3	114,100	114,100	118,008	118,008	112,980	113,175	110,149	107,271	87,164
RAsn4	131,929	131,929	105,528	105,528	108,296	108,324	105,657	102,499	71,378
RAsn5	144,510	144,510	79,332	79,332	110,070	110,683	106,965	104,222	87,458
RVal1	136,064	136,064	107,509	107,509	106,032	106,154	102,966	97,757	81,429
RVal2	113,040	113,040	105,789	105,789	104,285	104,383	101,329	98,417	78,400
RVal3	112,755	112,755	117,414	117,414	115,146	115,448	110,169	101,489	86,369
RVal4	133,738	133,738	120,965	120,965	119,455	119,798	116,950	114,593	97,847
RVal5	136,161	136,161	115,628	115,628	114,380	114,654	112,193	109,994	95,627
RA1C1	120,503	120,503	96,462	96,462	120,942	121,724	114,495	97,387	80,871
RA1C3	117,255	117,255	95,830	95,830	97,175	98,038	90,808	79,241	56,965

Sample	Raw forward	Raw reverse	Filtered forward	Filtered reverse	Denoised forward	Denoised reverse	Merged reads	Removed chimera	Only bacteria
RA1C5	159,665	159,665	100,523	100,523	96,267	96,969	89,338	77,198	55,687
RA1C7	126,545	126,545	97,968	97,968	88,819	89,300	83,894	71,707	56,967
RA2C1	148,187	148,187	123,356	123,356	98,209	98,825	92,066	77,515	72,296
RA2C3	125,017	125,017	100,114	100,114	66,896	67,188	60,363	50,549	44,434
RA2C5	118,774	118,774	99,378	99,378	89,018	89,685	83,304	69,293	60,050
RA2C7	121,843	121,843	90,670	90,670	99,024	99,744	92,947	76,090	66,617
RA3C1	145,070	145,070	100,437	100,437	91,700	92,093	86,607	74,673	55,627
RA3C3	126,052	126,052	68,530	68,530	106,703	107,176	100,672	84,357	76,282
RA3C5	130,668	130,668	91,490	91,490	89,238	89,799	83,353	69,091	56,873
RA3C7	142,098	142,098	101,650	101,650	99,433	100,140	94,719	77,256	53,372
RA4C1	137,985	137,985	93,565	93,565	104,606	104,879	99,544	81,334	57,180
RA4C3	130,545	130,545	108,891	108,891	86,418	86,724	84,174	64,645	26,308
RA4C5	135,007	135,007	91,449	91,449	97,713	97,953	95,163	74,242	27,006
RA4C7	124,729	124,729	101,411	101,411	85,458	85,804	82,811	62,307	30,198
RA5C1	120,999	120,999	106,090	106,090	98,126	98,421	93,306	76,999	38,010
RA5C3	127,492	127,492	87,037	87,037	90,164	90,412	86,761	68,401	27,806
RA5C5	108,186	108,186	98,509	98,509	88,520	88,813	84,805	67,666	24,472

Sample	Raw forward	Raw reverse	Filtered forward	Filtered reverse	Denoised forward	Denoised reverse	Merged reads	Removed chimera	Only bacteria
RA5C7	131,119	131,119	86,254	86,254	102,185	102,707	95,727	76,363	40,736
RA6C1	135,293	135,293	99,471	99,471	106,829	107,558	99,505	81,393	64,021
RA6C3	120,437	120,437	91,077	91,077	103,147	103,844	96,899	75,853	62,400
RA6C5	116,577	116,577	89,649	89,649	86,511	87,165	81,247	64,972	52,699
RA6C7	127,187	127,187	103,989	103,989	88,395	88,633	83,946	68,054	33,943
RA7C1	117,174	117,174	109,271	109,271	103,677	104,091	99,016	82,723	46,410
RA7C3	124,255	124,255	105,356	105,356	102,842	103,091	96,818	80,218	62,757
RA7C5	121,883	121,883	88,554	88,554	94,228	94,576	88,672	74,805	61,474
RA7C7	123,884	123,884	89,791	89,791	91,335	91,850	86,925	72,927	44,196
RA8C1	121,798	121,798	105,346	105,346	121,827	122,405	115,386	96,273	69,082
RA8C3	129,129	129,129	104,795	104,795	96,022	96,598	89,993	74,280	65,898
RA8C5	131,476	131,476	96,410	96,410	114,665	115,330	109,108	91,067	86,093
RA8C7	119,960	119,960	93,158	93,158	98,409	98,721	94,008	79,147	40,204
RA9C1	141,340	141,340	124,126	124,126	89,236	89,511	85,055	72,499	39,687
RA9C3	125,408	125,408	98,301	98,301	93,204	93,640	89,047	76,724	37,382
RA9C5	103,980	103,980	116,820	116,820	108,957	109,660	101,865	85,051	72,337
RA9C7	130,554	130,554	99,842	99,842	97,731	98,363	94,028	78,713	46,370

Sample	Raw forward	Raw reverse	Filtered forward	Filtered reverse	Denoised forward	Denoised reverse	Merged reads	Removed chimera	Only bacteria
RA10C1	127,937	127,937	90,760	90,760	94,179	94,781	88,399	73,631	66,691
RA10C3	141,858	141,858	94,849	94,849	93,573	93,942	86,969	73,053	56,293
RA10C5	145,908	145,908	111,725	111,725	98,083	98,601	91,667	74,290	57,554
RA10C7	138,014	138,014	99,431	99,431	95,842	96,322	89,385	73,298	60,726
R2-1	125,782	125,782	105,714	105,714	102,406	103,352	97,922	96,503	81,144
R2-2	132,838	132,838	113,236	113,236	108,893	109,718	102,092	99,506	85,518
R2-3	134,672	134,672	115,436	115,436	111,199	112,305	105,165	102,081	86,288
R3-1	148,398	148,398	126,531	126,531	120,245	121,666	110,644	105,279	91,159
R3-2	145,790	145,790	123,882	123,882	119,927	120,931	114,129	110,303	90,549
R3-3	165,602	165,602	141,295	141,295	134,745	136,049	125,020	119,331	100,716
R4-1	166,299	166,299	139,459	139,459	133,246	134,419	122,912	117,773	99,752
R4-2	155,487	155,487	131,574	131,574	125,737	127,235	116,361	108,461	92,081
R4-3	146,861	146,861	127,277	127,277	122,122	123,288	113,192	105,925	94,408
R5-1	145,850	145,850	124,847	124,847	119,138	120,549	110,844	108,240	88,955
R5-2	131,480	131,480	113,604	113,604	107,840	109,288	99,083	95,656	79,369
R5-3	177,838	177,838	154,842	154,842	148,259	149,935	138,191	132,486	107,034
R6-1	156,081	156,081	127,306	127,306	121,963	123,001	113,911	110,716	82,374

**Sample**	**Raw forward**	**Raw reverse**	**Filtered forward**	**Filtered reverse**	**Denoised forward**	**Denoised reverse**	**Merged reads**	**Removed chimera**	**Only bacteria**
R6-2	172,416	172,416	146,532	146,532	140,788	142,185	132,044	127,978	100,891
R6-3	151,801	151,801	130,286	130,286	123,747	125,052	113,768	110,627	83,863

Table S2. Pairwise PERMANOVA about Bray-Curtis distance about pathway prediction

Compare	Degree of freedom	Sum of squares	F model	R^2	*P*	P_{adj}	
Control vs Asn	1	0.008	0.446	0.053	0.608	0.678	
Control vs Asp	1	0.023	1.731	0.178	0.195	0.282	
Control vs Glu	1	0.005	0.269	0.033	0.750	0.785	
Control vs Val	1	0.033	3.213	0.287	0.075	0.146	
Control vs NH_4Cl	1	0.130	11.365	0.587	0.011	0.050	*
Control vs 1st	1	0.016	0.984	0.123	0.324	0.416	
Control vs 2nd	1	0.013	0.621	0.082	0.507	0.594	
Control vs 3rd	1	0.027	1.515	0.178	0.200	0.286	
Control vs 4th	1	0.396	11.360	0.619	0.015	0.058	
Control vs 5th	1	0.446	24.640	0.779	0.005	0.043	*
Control vs 6th	1	0.245	8.220	0.540	0.020	0.068	
Control vs 7th	1	0.139	6.551	0.483	0.029	0.079	
Control vs 8th	1	0.041	1.366	0.163	0.300	0.396	
Control vs 9th	1	0.153	5.543	0.442	0.051	0.114	
Control vs 10th	1	0.090	12.046	0.632	0.020	0.068	
Control vs Field	1	0.267	39.897	0.689	0.001	0.015	*
Asn vs Asp	1	0.008	0.410	0.049	0.715	0.772	
Asn vs Glu	1	0.002	0.093	0.011	0.900	0.907	
Asn vs Val	1	0.013	0.769	0.088	0.427	0.523	
Asn vs NH_4Cl	1	0.078	4.248	0.347	0.021	0.068	
Asn vs 1st	1	0.018	0.735	0.095	0.398	0.497	
Asn vs 2nd	1	0.008	0.278	0.038	0.709	0.771	
Asn vs 3rd	1	0.032	1.231	0.150	0.303	0.396	
Asn vs 4th	1	0.369	8.603	0.551	0.009	0.047	*
Asn vs 5th	1	0.419	16.030	0.696	0.006	0.043	*
Asn vs 6th	1	0.236	6.248	0.472	0.029	0.079	

Compare	Degree of freedom	Sum of squares	F model	R^2	*P*	P_{adj}	
Asn vs 7th	1	0.144	4.911	0.412	0.036	0.084	
Asn vs 8th	1	0.027	0.718	0.093	0.579	0.662	
Asn vs 9th	1	0.143	4.012	0.364	0.068	0.136	
Asn vs 10th	1	0.107	6.929	0.497	0.032	0.082	
Asn vs Field	1	0.176	17.923	0.499	0.001	0.015	*
Asp vs Glu	1	0.012	0.609	0.071	0.571	0.658	
Asp vs Val	1	0.002	0.144	0.018	0.817	0.842	
Asp vs NH_4Cl	1	0.057	4.226	0.346	0.054	0.118	
Asp vs 1st	1	0.039	2.067	0.228	0.147	0.227	
Asp vs 2nd	1	0.020	0.869	0.110	0.502	0.594	
Asp vs 3rd	1	0.064	3.096	0.307	0.108	0.184	
Asp vs 4th	1	0.416	11.148	0.614	0.017	0.062	
Asp vs 5th	1	0.486	23.609	0.771	0.009	0.047	*
Asp vs 6th	1	0.301	9.310	0.571	0.008	0.047	*
Asp vs 7th	1	0.206	8.685	0.554	0.015	0.058	
Asp vs 8th	1	0.044	1.366	0.163	0.264	0.359	
Asp vs 9th	1	0.196	6.513	0.482	0.019	0.068	
Asp vs 10th	1	0.167	16.881	0.707	0.021	0.068	
Asp vs Field	1	0.144	18.735	0.510	0.002	0.019	*
Glu vs Val	1	0.020	1.202	0.131	0.306	0.396	
Glu vs NH_4Cl	1	0.089	5.036	0.386	0.034	0.083	
Glu vs 1st	1	0.012	0.508	0.068	0.595	0.669	
Glu vs 2nd	1	0.004	0.155	0.022	0.840	0.853	
Glu vs 3rd	1	0.025	1.001	0.125	0.369	0.465	
Glu vs 4th	1	0.366	8.687	0.554	0.022	0.068	
Glu vs 5th	1	0.416	16.409	0.701	0.010	0.049	*
Glu vs 6th	1	0.231	6.219	0.470	0.028	0.079	
Glu vs 7th	1	0.136	4.765	0.405	0.040	0.092	

Compare	Degree of freedom	Sum of squares	F model	R^2	*P*	P_{adj}	
Glu vs 8th	1	0.026	0.692	0.090	0.588	0.666	
Glu vs 9th	1	0.139	3.995	0.363	0.062	0.130	
Glu vs 10th	1	0.097	6.627	0.486	0.022	0.068	
Glu vs Field	1	0.194	20.373	0.531	0.002	0.019	*
Val vs NH_4Cl	1	0.053	5.072	0.388	0.034	0.083	
Val vs 1st	1	0.052	3.452	0.330	0.101	0.178	
Val vs 2nd	1	0.028	1.488	0.175	0.181	0.265	
Val vs 3rd	1	0.079	4.683	0.401	0.061	0.130	
Val vs 4th	1	0.428	12.702	0.645	0.009	0.047	*
Val vs 5th	1	0.499	29.505	0.808	0.012	0.053	
Val vs 6th	1	0.316	11.038	0.612	0.009	0.047	*
Val vs 7th	1	0.223	11.144	0.614	0.016	0.060	
Val vs 8th	1	0.051	1.780	0.203	0.099	0.178	
Val vs 9th	1	0.209	7.905	0.530	0.013	0.055	
Val vs 10th	1	0.188	30.103	0.811	0.010	0.049	*
Val vs Field	1	0.138	22.130	0.551	0.001	0.015	*
NH_4Cl vs 1st	1	0.103	6.195	0.470	0.033	0.083	
NH_4Cl vs 2nd	1	0.072	3.497	0.333	0.015	0.058	
NH_4Cl vs 3rd	1	0.132	7.167	0.506	0.030	0.080	
NH_4Cl vs 4th	1	0.339	9.659	0.580	0.029	0.079	
NH_4Cl vs 5th	1	0.412	22.403	0.762	0.006	0.043	*
NH_4Cl vs 6th	1	0.292	9.690	0.581	0.011	0.050	*
NH_4Cl vs 7th	1	0.251	11.668	0.625	0.009	0.047	*
NH_4Cl vs 8th	1	0.064	2.104	0.231	0.090	0.165	
NH_4Cl vs 9th	1	0.204	7.305	0.511	0.009	0.047	*
NH_4Cl vs 10th	1	0.258	33.487	0.827	0.006	0.043	*
NH_4Cl vs Field	1	0.013	1.897	0.095	0.175	0.262	
1st vs 2nd	1	0.005	0.182	0.029	0.919	0.919	

Compare	Degree of freedom	Sum of squares	F model	R^2	*P*	P_{adj}	
1st vs 3rd	1	0.004	0.153	0.025	0.564	0.656	
1st vs 4th	1	0.276	6.170	0.507	0.087	0.164	
1st vs 5th	1	0.308	12.223	0.671	0.043	0.097	
1st vs 6th	1	0.152	3.915	0.395	0.062	0.130	
1st vs 7th	1	0.076	2.632	0.305	0.144	0.225	
1st vs 8th	1	0.018	0.451	0.070	0.746	0.785	
1st vs 9th	1	0.086	2.351	0.282	0.143	0.225	
1st vs 10th	1	0.048	3.750	0.385	0.028	0.079	
1st vs Field	1	0.185	21.681	0.561	0.002	0.019	*
2nd vs 3rd	1	0.014	0.473	0.073	0.676	0.741	
2nd vs 4th	1	0.278	5.640	0.485	0.066	0.134	
2nd vs 5th	1	0.314	10.547	0.637	0.035	0.084	
2nd vs 6th	1	0.166	3.809	0.388	0.066	0.134	
2nd vs 7th	1	0.094	2.801	0.318	0.132	0.215	
2nd vs 8th	1	0.010	0.218	0.035	0.730	0.780	
2nd vs 9th	1	0.092	2.247	0.272	0.239	0.328	
2nd vs 10th	1	0.073	4.199	0.412	0.133	0.215	
2nd vs Field	1	0.139	13.697	0.446	0.001	0.015	*
3rd vs 4th	1	0.259	5.521	0.479	0.117	0.196	
3rd vs 5th	1	0.276	10.105	0.627	0.025	0.076	
3rd vs 6th	1	0.125	3.042	0.336	0.102	0.178	
3rd vs 7th	1	0.051	1.657	0.216	0.363	0.461	
3rd vs 8th	1	0.022	0.539	0.082	0.654	0.723	
3rd vs 9th	1	0.065	1.689	0.220	0.268	0.361	
3rd vs 10th	1	0.026	1.725	0.223	0.075	0.146	
3rd vs Field	1	0.225	24.283	0.588	0.002	0.019	*
4th vs 5th	1	0.021	0.440	0.068	0.811	0.842	
4th vs 6th	1	0.052	0.867	0.126	0.306	0.396	

Compare	Degree of freedom	Sum of squares	F model	R^2	*P*	P_{adj}	
4th vs 7th	1	0.142	2.821	0.320	0.181	0.265	
4th vs 8th	1	0.193	3.182	0.347	0.090	0.165	
4th vs 9th	1	0.101	1.748	0.226	0.237	0.328	
4th vs 10th	1	0.246	7.144	0.544	0.100	0.178	
4th vs Field	1	0.475	29.329	0.633	0.004	0.036	*
5th vs 6th	1	0.034	0.838	0.123	0.454	0.546	
5th vs 7th	1	0.120	3.886	0.393	0.083	0.159	
5th vs 8th	1	0.224	5.448	0.476	0.104	0.179	
5th vs 9th	1	0.084	2.185	0.267	0.160	0.244	
5th vs 10th	1	0.229	15.402	0.720	0.031	0.081	
5th vs Field	1	0.573	61.698	0.784	0.001	0.015	*
6th vs 7th	1	0.027	0.596	0.090	0.473	0.564	
6th vs 8th	1	0.109	1.984	0.248	0.170	0.257	
6th vs 9th	1	0.016	0.301	0.048	0.829	0.848	
6th vs 10th	1	0.087	3.044	0.337	0.119	0.197	
6th vs Field	1	0.423	29.982	0.638	0.001	0.015	*
7th vs 8th	1	0.066	1.468	0.197	0.212	0.297	
7th vs 9th	1	0.009	0.216	0.035	0.734	0.780	
7th vs 10th	1	0.020	1.077	0.152	0.403	0.498	
7th vs Field	1	0.382	36.198	0.680	0.001	0.015	*
8th vs 9th	1	0.054	1.033	0.147	0.454	0.546	
8th vs 10th	1	0.071	2.461	0.291	0.138	0.221	
8th vs Field	1	0.120	8.468	0.332	0.002	0.019	*
9th vs 10th	1	0.048	1.844	0.235	0.207	0.293	
9th vs Field	1	0.304	23.000	0.575	0.001	0.015	*
10th vs Field	1	0.407	83.237	0.830	0.001	0.015	*

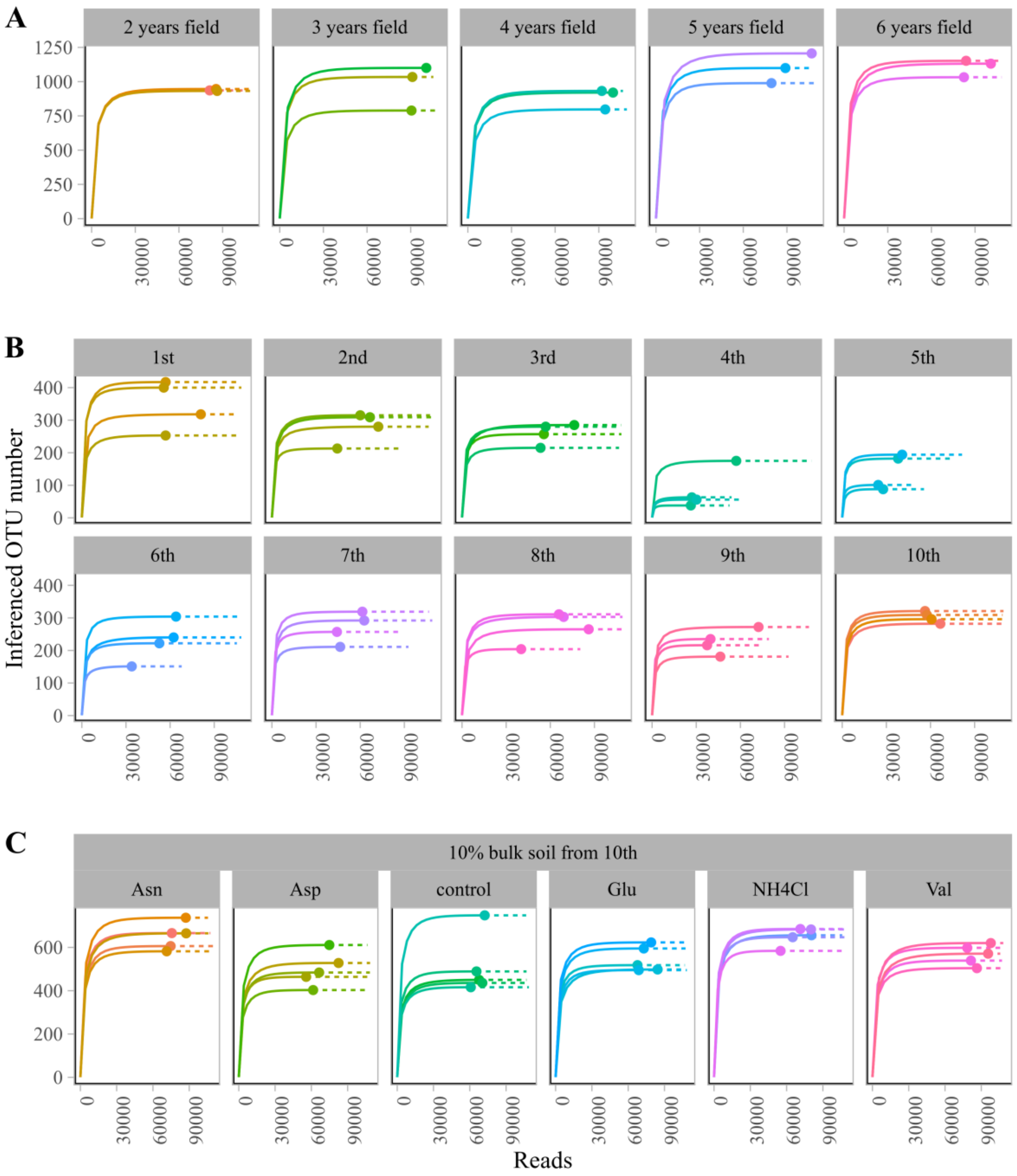

Figure S1. Rarefaction curve about OTU clustering. To reduce OTUs clustering different between field and previous pot results from the difference of error rate machine learning results in DADA2, OTUs of **(A)** the field samples in this study, **(B)** the serial replanting samples in pot, and **(C)** the different nitrogen treatment samples in the pot are clustered together. Reads and OTUs numbers of each sample are represented in the horizontal axis and vertical axis. When the number of reads increases, the number of OTUs converges without increasing, which is indicating that almost all bacteria have been sufficiently observed.
